# Role of nonresolving inflammation in hepatocellular carcinoma development and progression

**DOI:** 10.1038/s41698-018-0048-z

**Published:** 2018-02-23

**Authors:** Le-Xing Yu, Yan Ling, Hong-Yang Wang

**Affiliations:** 1 International Cooperation Laboratory on Signal Transduction, Eastern Hepatobiliary Surgery Institute, Second Military Medical University, Shanghai, 200438 China; 2 National Center for Liver Cancer Research, Shanghai, China; 3 Ministry of Education (MOE) Key Laboratory on Signaling Regulation and Targeting Therapy of Liver Cancer, Shanghai, 200438 China; 4 State Key Laboratory of Oncogenes and Related Genes, Shanghai Cancer Institute, Renji Hospital, Shanghai Jiaotong University School of Medicine, Shanghai, 200032 China

## Abstract

Hepatocellular carcinoma (HCC) has become a leading cause of cancer-related death, making the elucidation of its underlying mechanisms an urgent priority. Inflammation is an adaptive response to infection and tissue injury under strict regulations. When the host regulatory machine runs out of control, nonresolving inflammation occurs. Nonresolving inflammation is a recognized hallmark of cancer that substantially contributes to the development and progression of HCC. The HCC-associated inflammation can be initiated and propagated by extrinsic pathways through activation of pattern-recognition receptors (PRRs) by pathogen-associated molecule patterns (PAMPs) derived from gut microflora or damage-associated molecule patterns (DAMPs) released from dying liver cells. The inflammation can also be orchestrated by the tumor itself through secreting factors that recruit inflammatory cells to the tumor favoring the buildup of a microenvironment. Accumulating datas from human and mouse models showed that inflammation promotes HCC development by promoting proliferative and survival signaling, inducing angiogenesis, evading immune surveillance, supporting cancer stem cells, activating invasion and metastasis as well as inducing genomic instability. Targeting inflammation may represent a promising avenue for the HCC treatment. Some inhibitors targeting inflammatory pathways have been developed and under different stages of clinical trials, and one (sorafenib) have been approved by FDA. However, as most of the data were obtained from animal models, and there is a big difference between human HCC and mouse HCC models, it is challenging on successful translation from bench to bedside.

## Introduction

Liver cancer is the sixth most common cancer and the second leading cause of cancer death worldwide.^1^ About half of the cases and deaths occurring in China, where it is the fourth most diagnosed cancer and the third cause of cancer-related death, with an estimated 466,100 new cases and 422,100 deaths in 2015.^2^ The prognosis for liver cancer is unfavorable, showed by the 10.1% of age-standardized 5-year relative survival in China.^3^ In the present review, we will focus on the most common histologic type of liver cancers-hepatocellular carcinoma (HCC), which represents 85–90% of primary liver cancers.^4^ Epidemiologic studies showed that HCC has several specific epidemiologic features including dynamic temporal trends, marked variations among geographic regions, racial and ethnic groups, gender disparity, and the presence of environmental potentially preventable risk factors.^5^ HCC predominantly arises as the end stage of liver diseases, persistent inflammation with hepatitis B or C virus (HBV, HCV) infections, alcoholic liver disease, and nonalcoholic fatty liver disease being the current leading causes.^4^ Other risk factors include biliary diseases, metabolic disorders, drugs, toxins, and genetic conditions such as hereditary hemochromatosis and 1-antitrypsin deficiency.^5^ Most of the risk factors lead to the formation and progression of liver cirrhosis, which is present in most (>80%) of HCC patients.^6^ Due to inadequate understanding of the molecular features and genomic traits, lack of suitable biomarkers for early detection, and resistant to chemotherapies, current treatment for HCC remains a big challenge.^7^ To complicate matters further, aggressive treatment strategies for liver cancer are frequently limited because of the underlying liver cirrhosis and severely compromised liver function.^7^ Sorafenib is the only drug approved by the FDA for the treatment of advanced HCC.^8,9^ Nevertheless, only moderate improvement of survival, a number of adverse side effects, and high costs underscore the need for other novel therapeutics as well as preventive approaches for HCC.^10^ Thus, the elucidation of underlying mechanisms of HCC is now becoming an urgent priority.

Inflammation is an adaptive response to infection and tissue injury, characterized by the blood vessel reaction, immune cell recruitment, and release of molecular mediators, all of which aimed at fighting against the pathogens or harmful stimuli, repairing damaged tissue, and restoring homeostasis.^11^ In this sense, a successful inflammatory response results in the elimination of the assaulting agents followed by a resolution and repair phase. The fine orchestration of cells and soluble factors–not only the expression or extinction of certain critical mediator but also their tuning and timing–during inflammation ensures the resolution of inflammation. However, under certain circumstances when the inflammatory stimuli persists or the regulatory mechanism runs out of control (e.g., subnormal inflammatory response, prolonged or excessive response, inadequate production of resolution mediators, failed phenotypical switch in macrophage and T-cell populations, as well as infiltration by immune-suppressive cells),^12^ the nonresolving inflammation occurs and may have pathological consequences, such as autoimmunity, fibrosis, metaplasia and/or tumor growth.^11^ The recently marked advance in the cancer study has well established the functional relationship between inflammation and cancer,^13^ and tumor-promoting inflammation has been considered as a hallmark of cancer.^14^

HCC represents a classic paradigm of inflammation-linked cancer, as more than 90% of HCCs arise in the context of hepatic injury and inflammation.^4^ The risk factors of HCC usually elicit a nonresolving inflammation response characterized by infiltration of macrophage and immature myeloid cell and dysregulated production of cytokines, resulting in the perpetuation of the wound-healing response and leading to the sequential development of fibrosis, cirrhosis, and eventually HCC. In the premalignant stage of hepatocarcinogenesis is a multistage and long-term process, chronic activation of inflammatory signaling pathways result in the generation of reactive oxygen species (ROS) and reactive nitrogen species (NOS). In concert with chronic compensatory hepatocyte regeneration and proliferation-induced mutagenesis, chronic ROS and NOS exposure sets the stage for HCC development. In parallel, inflammatory cells (and other stromal cells) within the premalignant environment produce a vast array of cytokines, growth factors, chemokines, prostaglandins, and proangiogenic factors, contributing to an environment that supports the transformation of hepatocyte but also promotes their survival through activation of anti-apoptotic pathways and inhibition of immune surveillance.^15^ In support of this, there are evidences indicating that the use of nonsteroidal anti-inflammatory agents decrease the incidence and/or recurrence of HCC.^16–18^ In the established HCC, inflammatory cells and the factors they produced influence multi-aspects of the growth and behavior of tumor cells and also modulate therapeutic effects by cooperating with other types of stromal cells. In the present review, we will focus on the chronic inflammation during hepatocarcinogenesis, and discuss the triggers of inflammation and how inflammation contributes to the hallmarks of HCC.

## Pathways that trigger nonresolving inflammation during hepatocarcinogenesis

**Fig. 1:**
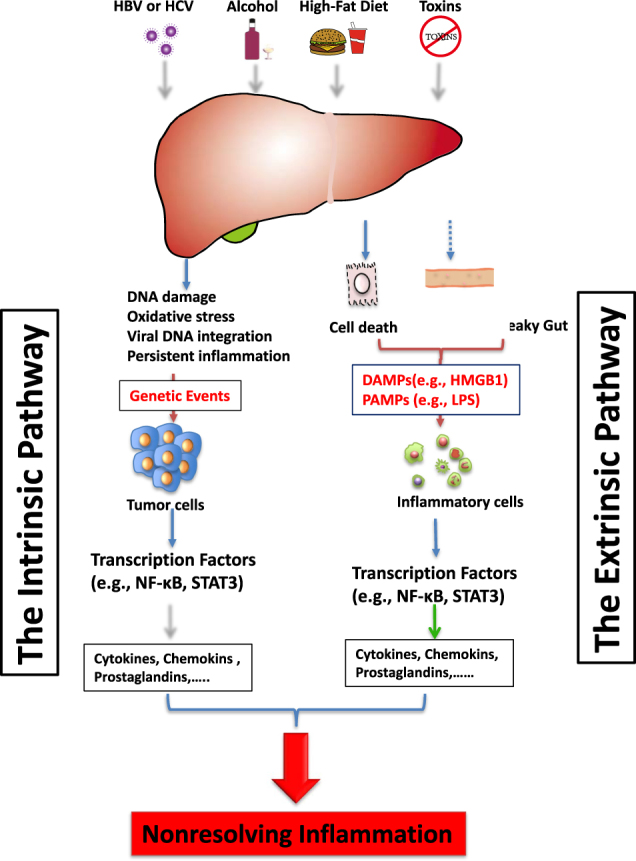
The extrinsic and intrinsic pathways that trigger nonresolving inflammation during hepatocarcinogenesis. The extrinsic pathway is driven by exogenous factors (e.g., the PAMPs from pathogens or DAMPs from dead cells), which are able to trigger a persistent inflammatory response by engaging the receptors expressed in the inflammatory cells and establish an inflammatory condition that increase cancer risk. On the other hand, the intrinsic pathway is induced by alterations in cancer-associated genetic factors (e.g., mutation of either oncogenes or tumor suppressor genes), which activate the expression of inflammation-related program. Both of these pathways activate transcription factors (e.g., NF-κB, STAT3) that coordinate the production of inflammatory mediators, including cytokines, chemokines, ROS, NOS, prostaglandins and so on, generating a pro-tumoric inflammatory microenvironment in the liver

Nonresolving inflammation results from the persistence of initiating stimuli or deficiencies in the inflammation-resolving mechanisms. Its key features include immune cells (tumor-associated macrophages (TAM), immature myeloid cells, T cells) infiltration; the presence of inflammatory mediators; the imbalance of pro- (such as TNF-α, IL-6, IL-1) vs. anti-inflammatory cytokines (such as IL-10, IL-12, TGFβ); and the occurrence of angiogenesis and tissue remodeling. Two distinct pathways that causing nonresolving inflammation during cancer development have been described.^19^ In the extrinsic pathway, exogenous factors (e.g., the PAMPs from pathogens or DAMPs from necrotic cells) activate the inflammatory responses after sensed by the inflammatory cells and establish an inflammatory condition that increase cancer risk. In the intrinsic pathway, genetic events (e.g., oncogenes, tumor suppressor genes) causing neoplastic transformation activate the expression of inflammation-related programs guiding the construction of an inflammatory microenvironment. These two pathways can both activate transcription factors (e.g., NF-κB, STAT3), which are key orchestrators of the inflammatory response such as the production of cytokines, chemokines (Fig. 1). Nonresolving inflammation is a major driver of HCC. Similarly, hepatocarcinogenesis is accompanied with these two types of inflammation. Notably, the fact that HCC usually develops as the end stage of chronic liver diseases (CLD) makes the connection between HCC and inflammation more intense than in other cancers. Moreover, as the liver is closely connected with the gut both anatomically and functionally, the gut-derived PAMPs may play more important roles in the inflammation process than in other cancers.

### The extrinsic pathway

The extrinsic signals are mainly sensed by inflammatory cells expressed PRRs that recognize structures conserved among microbial species as well as endogenous molecules released from damaged cells, which are called PAMPs and DAMPs, respectively.^20^ Currently, four major classes of PRR families have been identified, including transmembrane proteins such as the toll-like receptors (TLRs) and C-type Lectin receptors (CLRs), cytoplasmic proteins such as the retinoic acid-inducible gene (RIG)-I-like receptors (RLRs) and NOD-like receptors (NLRs).^20^ The recognition of PAMPs (e.g., proteins, nucleic acids, lipids and carbohydrates, derived from foreign microorganisms) and DAMPs (such as high-mobility group box 1 (HMGB1), ATP, and heat-shock proteins) by PRRs lead to the transcriptional expression of inflammatory mediators that coordinate the elimination of pathogens and infected cells.^20^ Despite the crucial role of PRRs in activating innate and adaptive immune responses, inappropriate activation of PRRs can lead to prolonged inflammation and even to autoimmune and inflammatory diseases. In the liver, PRRs are broadly expressed in a variety of cell types,^21,22^ both the nonparenchymal (immune cells, vascular cells, stellate cells, etc.) and parenchymal cells, and involved in the liver function maintenance and liver diseases development. The TLR family is one of the best-characterized PRRs in the progression of chronic live diseases. Ligands binding to TLRs activate multiple proinflammatory signaling cascades linking chronic inflammation and HCC, including nuclear factor (NF)-κB, c-Jun N-terminal kinase/AP1, extracellular signal regulated kinase(ERK) and p38, as well as the interferon pathway.

#### PAMPs and nonresolving inflammation—the gut-liver axis

The anatomy of the liver provides its close interaction with the gut where nutrients and the microbiome (and their products) contribute to the maintenance of a healthy metabolism in liver. The liver functions as a firewall to filter out the commensal bacteria and their products.^23^ However, this function is attenuated in CLD,^23,24^ making increased bacterial translocation, a hallmark of CLD, which not only contributes to characteristic infectious complications but also generates a chronic inflammatory state in the liver.^25^ Among all the PAMPs/PRR signaling pathways, the role of lipopolysaccharide (LPS)/TLR4 signaling in CLDs has been emphasized by many studies. Clinically, high levels of LPS occur in patients with cirrhosis because of an increase in intestinal mucosal permeability and bacterial translocation.^26^ In animal models, gut sterilization using non-absorbable antibiotics or genetic *TLR4* inactivation suppressed liver inflammation, fibrosis, and HCC.^21,22,27^ The LPS/TLR4 signaling pathway activation in Kupffer cells led to TNFα- and IL-6-dependent hepatocyte compensatory proliferation and reduction of oxidative and apoptotic stress.^21^ TLR4 signaling in hepatic stellate cells led to the transcription factor NF-κB p65 subunit nuclear translocation, and upregulation of the epiregulin (an epidermal growth factor (EGF) family member) during the early stages of DEN/CCl4 carcinogenesis, whereas it reduces hepatocyte apoptosis by NF-κB activation during the late stages of hepatocarcinogenesis.^22^ In a DEN-induced rat HCC model, restoration of gut homeostasis using probiotics led to reduced inflammation, fibrosis and HCC, further confirming the functional link between gut and liver during hepatocarcinogensis.^26^

#### The DAMPs and nonresolving inflammation—the role of HMGB1

DAMPs represent a large range of chemically unrelated mediators such as HMGB1, S100 proteins, heat-shock proteins, ATP and calreticulin that are retained inside the cell in the healthy state and only released or surface exposed following stress or cell death, thus allowing the host to sense and react to damage via specific DAMP receptors.^28^ They function as either adjuvant or danger signals for the immune system, and potently trigger sterile inflammation. DAMPs were mainly released by necrotic cells that were induced by liver injury, immune response, lack of nutrition and oxygen in the fast-growing tumors, as well as cancer therapy. Recent evidence suggests that DAMPs can also be released by specific forms of programmed cell death such as necroptosis and immunogenic cell death following anti-cancer therapies.^28,29^

DAMPs elicit significant inflammation by activating PRRs. One of intensively studied DAMPs is HMGB1, which is a highly conserved non-histone nuclear protein that facilitates binding of regulatory proteins to DNA and typically enhances transcriptional activation.^30^ When released, HMGB1 can be recognized by several PRRs, such as TLR2, TLR4, TLR9, and RAGE, and activate a number of inflammatory pathways.^31^ It has been shown that HMGB1-mediated proinflammatory cytokine production plays crucial roles in liver ischemia/reperfusion (I/R) injury, viral hepatitis, CLDs such as NAFLD, NASH and liver fibrosis, as well as HCC development.^32^ HMGB1 released by hypoxic cells was shown to promote HCC cell invasion and metastasis by activating TLR4- and RAGE- pathway.^33,34^ HMGB1 receptor RAGE deficiency suppresses carcinogenesis in inflammation-induced liver cancer and decreased oval cell activation, while HMGB1 promoted oval cell proliferation.^35^ Moreover, HMGB1 promotes the growth of hepatocellular carcinoma in concert with mitochondrial DNA via activation of TLR9.^36^ Ectopic expression of HMGB1 in the liver exacerbates DEN-induced liver injury and cytokine production, while HMGB1 release inhibitor ethyl pyruvate mitigated DEN-induced liver injury and tumorigenesis after DEN treatment in rats.^37^ HMGB1 is important for the recruitment of immune cells. For example, hepatic HMGB1 triggered the recruitment of neutrophils, subsequent inflammation, and amplification of liver injury through RAGE.^38^ In melanoma, HMGB1 released by ultraviolet-radiation damaged epidermal keratinocytes also was showed to initiate, via a TLR4-dependent manner, the recruitment and activation of neutrophils that promotes angiotrophism and metastasis.^39^ In acetaminophen-induced liver injury, the HMGB1-TLR4-IL-23 pathway in macrophages induces the generation of IL-17-producing γδT cells, which mediates neutrophil infiltration and damage-induced liver inflammation.^40^ HMGB1 may exert a chemotactic effect on T cells, as it is reported that HMGB1 promotes the recruitment and activation of intratumoral T cells, which in turn recruit tumor-promoting macrophages in prostate cancer.^41^ But this effect of HMGB1 in HCC needs to be further investigated. HMGB1 also promoted proliferation and migration of HSCs, suggesting that HMGB1 might be an effective target to treat liver fibrosis.^42^ Collectively, these data clearly show that HMGB1 promotes hepatocarcinogenesis by inducing or maintaining chronic inflammation. Moreover, the evidence that interaction between HMGB1 and the receptor TIM-3 expressed on tumor-associated dendritic cells is critical for evasion of the immune system by tumor cells in response to DNA-containing vaccines and chemotherapy^43^ indicating that HMGB1 also promotes carcinogenesis through immunosuppressive pathways.

### The intrinsic pathway

Many genetic events that cause neoplasia are shown to be responsible for initiating an inflammatory signaling cascade and contribute significantly to the tumor-promoting inflammatory microenvironment. Genetically transformed (such as activation of oncogenes, inactivation of tumor-suppressor genes, or chromosomal rearrangement^44^) epithelial cells can produce inflammatory mediators that recruit immune cells to the tumor enabling the buildup of a favoring microenvironment. For instance, the oncogenic *Ras* was shown to transcriptionally upregulate proinflammatory cytokine such as interleukin-8 (IL-8), IL-6, CXCL-1, and CXCL-2, contributing to tumorigenesis-supporting milieu.^45^ Oncoprotein *Src* activation also led to the malignant transformation of breast epithelial cell by activation of the IL-6 signaling pathway.^46^ For HCC, frequent alterations are known to occur in key cancer genes/pathways such as *TP53*, *WNT*, and *CTNNB1* (encoding *β­catenin*). In a β-catenin-induced hepatocarcinogenesis model, β-catenin activation in hepatocytes triggered a smoldering inflammation through the control of both pro- and anti-inflammatory programs that buildup a protumorigenic microenvironment with a low grade of chronic inflammation.^47^ TP53 is involved in the inflammatory response as p53 and NF-κB antagonizes each other’s function,^48^ whereas mutant p53 has been reported to augment and prolong NF-κB activation in cultured cells as well as in mice^49,50^ and initiates a chronic inflammatory condition in animals exposed to tissue damage, thus promote the development of the inflammation-associated colorectal cancer.^51^ In the liver, mutant p53^R172H^ were shown to cause spontaneous liver inflammation, steatosis, and fibrosis in vivo in combination with IL27RA deficiency.^52^ However, liver-specific deletion of p53 led to the tumor formation without affecting the inflammation in the liver.^53^ Some data showed that wild-type p53 accumulation in hepatocyte promote liver fibrosis by inducing the cytokine CTGF production,^54^ or promote chronic inflammation by inducing HMGB1 release.^37^ Thus, the roles of p53, especially some commonly mutated forms, in liver inflammation need further investigations. The tumor suppressor PTEN can greatly affect inflammation in the liver and HCC formation.^55^ Specific deletion of PTEN in hepatocytes leads to fatty-acid accumulation, inflammatory cells recruitment and HCC development, which is partially due to AKT2, as disruption of both AKT2 and PTEN rescues this phenotype.^56^ Mutations were also frequently found in some inflammatory pathways such as NF-κB, IL-6, TGFβ, and PDGF signaling pathways by recent sequencing approach.^57^ Indeed, acquirement of autocrine IL-6 signaling rendered the selective advantage to the HCC progenitor cell for the in vivo growth and malignant progression.^58^

## Key orchestrators that connect nonresolving inflammation and hepatocarcinogenesis

### NF-κB signaling pathway

The NF-κB refers to a family of transcriptional factors that recognize the κB sequence motif. During cancer development, NF-κB, which can be activated by inflammatory stimuli or oncogenic events, is a master regulator of proinflammatory, proliferative, and prosurvival genes,^13^ thus exerts key roles in the inflammatory response and cancer development. NF-κB has crucial roles in the regulation of inflammation in the liver-it is activated in almost all the CLDs; its activation regulates multiple essential functions of the liver cells, and inactivation of NF-κB in animal models results in deregulated liver homeostasis and wound-healing responses.^59^ In the parenchymal cells, the role of NF-κB during hepatocarcinogensis is controversial. In the multidrug resistance protein 2 (MDR2)-deficient mouse, TNFα produced by endothelial cells and inflammatory cells induced NF-κB activation in hepatocytes. This process was required for progression to HCC but not for hepatocyte transformation.^60^ In another inflammatory HCC mouse model, where the cytokines lymphotoxin α and/or lymphotoxin β was overexpressed in the liver, NF-κB inhibition by IKK2 deletion in hepatocytes significantly inhibited hepatocarcinogenesis.^61^ Consistent with the tumor-promoting effects of NF-κB raised by these studies, a report showed that in a nonalcoholic steatohepatitis mouse model, TNFα produced by infiltrating macrophages activated TNF receptor 1 (TNFR1)-NF-κB signaling in hepatocytes resulting in tumor growth.^62^ However, some studies showed that hepatocyte NF-κB signaling suppressed liver cancer development rather than promoting. NF-κB inhibition in hepatocytes by IKK2 deletion resulted in increased HCC burden in the DEN-induced hepatocarcinogenesis model.^63–65^ Similarly, inhibition of NF-κB activation by nemo deficiency (the regulatory IKK subunit) in hepatocytes led to spontaneous tumor formation in mice.^66,67^ This discrepancy may reflect the cellular specificity of NF-κB, the timing of NF-κB activation as well as characteristics of liver damage. NF-κB is also activated during the cellular response to DNA damage^68^ thereby promote the survival and proliferation of mutated cells. Interestingly, inhibition of NF-κB by IKK2 deficiency in the later stages of hepatocarcinogenesis also accelerated the development of HCC by promoting proliferation/survival of DEN-initiated hepatocytes and augmenting STAT3 activation.^65^ This data suggests that hepatocyte IKK2/ NF-κB prevented hepatocarcinogensis through inhibiting liver damage and hepatocyte proliferation. In contrast to the inconsistent data of parenchymal NF-κB in hepatocarcinogenesis from different reports, NF-κB in myeloid cells were shown to promote HCC development by activating target genes expression, including a panel of inflammatory cytokines and growth factors such as TNFα and IL-6. In the DEN model, where inhibition of IKKβ/NF-κB signaling in hepatocytes was shown to promote HCC development, additional deletion of IKKβ in liver myeloid cells diminished the production of proinflammatory cytokines, reduced liver compensatory proliferation, and strongly inhibited DEN-induced hepatocarcinogenesis.^63^ The activation of NF-κB in the myeloid cells was shown to be dependent on the release of IL-1α by the dying hepatocytes.^69^ Moreover, NF-κB apparently polarizes macrophages toward the alternatively activated M2 phenotype, which are tumor-promoting and immunosuppressive.^70^

### STAT3 pathways

HCC typically develops in severely perturbed livers, which display profound architectural and functional alterations. Growth factors and their receptors play crucial roles in governing hepatic architecture and function by transmitting proliferative, survival, and homeostatic signals. A wide range of input signals are integrated by growth factor receptors (usually tyrosine kinases or coupled with tyrosine kinases), leading to cell proliferation and survival, as well as cellular differentiation, adhesion, migration, and metabolism^71^ by activating a few major intracellular signaling cascades. Among them, the activation of signal transducer and activator of transcription (STAT) family members, in particular STAT3, is closely linked to inflammatory processes in liver tumorigenesis.^72^ Activated STAT3 was detected in ~60% of human HCC specimens and STAT3-positive tumors were more aggressive.^65^ In the experimental systems, hepatic STAT3 activation is crucial for hepatocarcinogenesis in mice^65^ and the maintenance of cancer stem cells (CSCs).^73^ Although the inducers for STAT3 activation in human HCC have not been fully understood, it is most likely activated by cytokines and growth factors produced within the tumor microenvironment (TME). Indeed, the expression of IL-6, one of the major STAT3-activating cytokines, is elevated in human liver diseases and HCC.^74^ In animal models, myeloid cell-derived IL-6 was shown to be a general mechanism for progenitor cell-induced HCC,^58^ the gender disparity^75^ and obesity-associated hepatocarcinogenesis.^76^ IL-6/STAT3 signaling was also shown to induce an increase in the CD133^+^ cancer progenitor population in vitro.^77^ IL-22 that is produced by HCC-infiltrating inflammatory cells is another cytokine, which promotes HCC development by activating STAT3 signaling pathway.^78^ Consistently, hepatocyte-specific overexpression of IL-22 resulted in increased hepatocarcinogensis by promoting cell survival as well as proliferation without significantly affecting liver inflammation.^79^ Other activators for STAT3 in HCC include LIF (leukemia inhibitory factor),^80^ miR24 or miR-629.^81^ Mechanistically, STAT3 promotes cell proliferation by activating the expression of its target genes, such as the cell cycle regulators cyclin D1, cyclin D2, and cyclin B, as well as the oncogene MYC, while the anti-apoptotic genes such as BCL2 and BCL-X_L_ mediate the prosurvival effects of STAT3.^82^ In the immune cells, constitutive activation of STAT3 has been demonstrated in tumor-infiltrating inflammatory cells and was associated with M2 polarization of macrophage,^83^ thus counteracting anti-tumor immune response.

### Crosstalk between NF-κB and STAT3 pathways

NF-κB and STAT3 both control the expression of a large number of downstream genes that control cell proliferation, survival, stress responses, and immune functions. Some of the target genes of NF-κB and STAT3 overlap. In addition, the two transcription factors are engaged in positive crosstalk.^84^ In a mouse DEN model, IL-1α released from DEN-damaged hepatocytes led to the IL-6 production in kupffer cells via a NF-κB-dependent manner.^63,69^ IL-6 thus activated STAT3 in hepatocytes and induced expression of STAT3 target genes that are critical for compensatory hepatocyte proliferation and liver tumorigenesis.^65,75^ Meanwhile, STAT3 can contribute to NF-κB activation by prolonging p65 nuclear retention.^85^ However, these two transcription factors are also engaged in negative crosstalk within HCC cells via the ROS-mediated oxidizing protein tyrosine phosphatases (PTPs) that dephosphorylate activated JAK2/STAT3.^65^ Therefore, both these positive and negative crosstalks complicate the inflammation network during hepatocarcinogenesis, and need to be elucidated when targeting these pathways for therapeutic purpose.

## Mechanisms by which inflammation promotes hepatocarcinogenesis

**Fig. 2:**
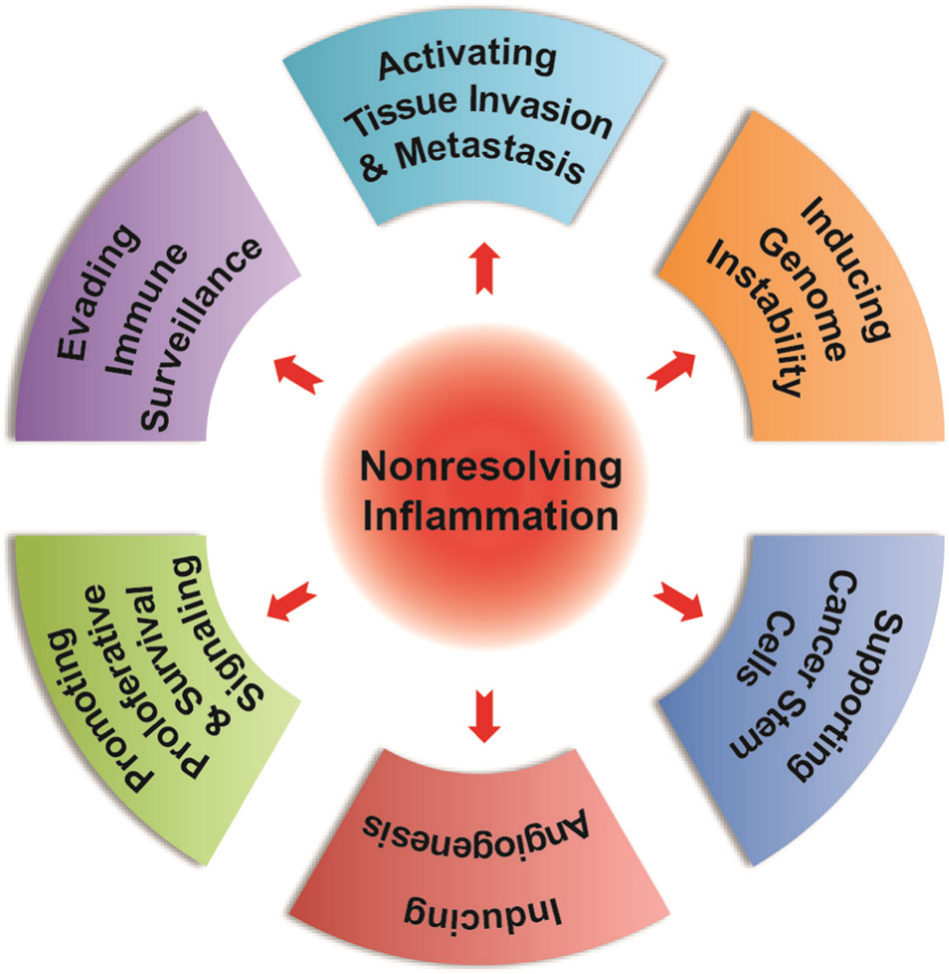
Nonresolving inflammation contribute to the hallmark of hepatocellular carcinoma

Nonresolving inflammation is considered as an enabling characteristic for its contribution to the acquisition of other hallmark capabilities of cancer.^14^ Here, we enumerate the mechanisms demonstrably attributed to the inflammation during hepatocarcinogenesis (Fig. 2).

### Promoting proliferative and survival signaling

The infiltrating immune cells supply direct and indirect mitogenic mediators that stimulate proliferation of tumor cells as well as other stromal cell types in their vicinity. Notable examples include the EGFR ligands, IL-6, TNFα, FGFs, TGFβ, HGF.^86^ In addition, the inflammatory cells can produce metalloproteinases (MMPs) that selectively cleave extracellular matrix (ECM) thereby modify the structure and function of the HCC microenvironment. Degradation of ECM not only result in the remove of physical restraint for the tumor growth and clonal expansion, but also modulate the release of sequestered growth factors, generate bioactive cleavage products, or cleave cell surface receptors,^15^ all of which provide the pro-proliferative signals for tumor cells. Moreover, ECM degradation disables the growth-suppressing adhesion complexes maintaining hemostasis thereby helps the tumor cells evade growth suppression.^87^ Infiltrating immune cells may also provide survival signals to HCC cells that limit the impact of cancer cell death programs on tumor progression, which are triggered by a variety of tissue protective and therapy-induced mechanisms. For instance, in HCC xenografts, sorafenib increased TAM infiltration via induction of CXCL12. Depletion of TAMs potentiated the inhibitory activity of sorafenib on angiogenesis, primary tumor growth, and metastasis.^88^

### Inducing angiogenesis

The growth of HCCs depends on their ability to form new blood vessels a process called angiogenesis for the nutrient and oxygen supply.^89^ There is a tight interaction between inflammatory cells and vascular cells in the tumor TME. The infiltrating inflammatory cells produce a diverse assortment of factors that influence endothelial cell behavior, while endothelial cells recruit leukocytes by expressing a repertoire of adhesion molecules. The soluble mediators produced by inflammatory cells implicated in regulating angiogenesis include cytokines (VEGF, bFGF, TNFα, TGFβ, platelet-derived growth factor, placental growth factor), chemokines (CXCL12, IL-8/CXCL8), MMPs (e.g., MMP-2, -7, -9, -12, and -14), and so on. All of these effectors are capable to regulate vascular cell survival, proliferation and/or motility, culminating in tissue remodeling, and new vessel formation.^87^

TAM presents the major component of leukocytes that infiltrating in the TME and is an important resource of the key proangiogenic factor VEGF. Clinical data has shown a positive correlation between the frequency of TAMs and the density of microvessels.^90^ Macrophage depletion by zoledronic acid or clodronate-encapsulated liposomes, in combination with sorafenib, further reduced tumor growth, lung metastasis, and tumor angiogenesis.^88^ Moreover, VEGF may be released from the ECM via MMP-9 (which was produced by TAM)-mediated proteolysis,^91^ thus providing an alternative, but still VEGF-dependent route for promoting angiogenesis. Similarly, TAM production of the VEGF family member PIGF stimulates angiogenesis in HCC^92^ and, thus, TAMs may present a mechanism for acquiring resistance to anti-VEGF-A/VEGFR therapies.^87^ TAMs are preferentially attracted to hypoxic areas in tumor. Hypoxia-induced factor-α in TAMs is essential for TAM infiltration and activation in vivo.^93^ Notably, one of the TAM subsets, the angiopoietins receptor TIE2 (tyrosine kinase with Ig and EGF homology domains 2) expressing macrophage, has relatively strong proangiogenic activity. TIE2^+^ TAMs selectively migrate toward angiopoietin 2 released by ECs especially in hypoxic tumor areas.^94^ In HCC, the frequency of TIE2^+^ TAMs was positively correlated with microvessel density and may serve as a diagnostic marker for HCC.^95^

Other types of infiltrating inflammatory cells in TME also contribute to pro-tumoric angiogenesis. Neutrophils were found enriching predominantly in peritumoral stroma of HCC tissues and were the major source of MMP-9 that stimulated proangiogenic activity in hepatoma cells.^96^ Tu et al.^97^ reported that intratumoral-infiltrated mast cells were the main source of the proinflammatory cytokine IL-17 and their number was positively correlated with microvessels density in HCC, suggesting a proangiogenic role of mast cells.^97^ The myeloid-derived suppressor cells (MDSCs) are important components of immune cells in the TME, and they promot tumor angiogenesis directly through producing high levels of MMP-9.^98^

### Evading immune surveillance

The liver is heavily populated with innate and adaptive immune cells. These cells play important roles in eliminating premalignant or malignant hepatocytes as shown in several animal models.^99–102^ However, in chronically inflammatory liver, there are several ways that help tumor cells evading the immune surveillance during hepatocarcinogenesis. (i) The phenotypic switch of macrophage from anti-tumoral M1 to tumor-promoting and immunosuppressive M2 phenotype. This switch can be driven by various cytokines and signals expressed in the TME, which can be provided by either stromal cells or tumor cells, such as IL-10, glucocorticoid hormones, apoptotic cells, and immune complex. A recent report showed that deletion of p53 in hepatic stellate cells led to the macrophage M2 polarization thus avoided immune elimination and resulted in accelerated HCC development in a DEN-induced HCC model.^100^ Moreover, liver macrophages in the chronic inflammation condition mainly derived from the circulating monocyte. Blockage of maturation of recruited myeloid precursors by HCC cells promotes the growth of HCC through inhibition of NK-cell activity.^99^ (ii) Downregulation of T-cell effector functions due to increased inhibitory checkpoint signaling and/or decreased co-stimulatory signaling. Upregulated expression of PD-1, CTLA-4, T-cell immunoglobulin domain and mucin domain 3 (Tim3) on lymphocytes and their corresponding ligands PD-L1/L2, B7-1/7-2, galectin3 on Kupffer cells, endothelial cells, monocytes as well as tumor cells has been reported both in chronic hepatitis and HCC^103^ Meanwhile, significant reductions of B7-1 and B7-2 expression have been identified on HCC cells resulting in a decrease of B7/CD28-mediated activation of effector T cells.^104^ (iii) Increased recruitment of immune-suppressive cells, such as Treg cells,^105^ invariant nature killer T cells,^106^ MDSC^107^ and TAMs,^108^ as well as decreased CD4^+^ T cells^101^ have been reported in HCC. A new subset of immune-suppressive cells in HCC patients called regulatory DCs have been identified, which suppressed T-cell activation through IL-10 and indoleamine 2,3-dioxygenase (IDO) production, which was CTLA-4-dependent.^109^ (iv) Decreased recognition of malignant cells by the immune system as a result of the failure of HCC-associated antigen processing and presentation by the antigen-presenting cells (APC).^110^ (v) Production of immunosuppressive cytokines such as IL-10, TGFβ, chemokines, and so on.^111^ A unique innate immunity signature within the TME includes an increase in immunosuppressive cytokines (IL-4, IL-5, IL-8, and IL-10) accompanied by suppression of immune-activating cytokines (IL-1, TNFα), and IFNγ has been reported to promote HCC metastasis.^112^ High-serum IL-10 levels were shown to be associated with poor prognosis in HCC patients.^113^

### Supporting cancer stem cells

Recent years, more and more evidence has supported the existence of CSCs or tumor-initiating cells (TICs) within tumors including HCC. These cells have important roles in the pathologic manifestation of cancer, variably affecting tumor initiation, proliferation and survival, metastatic progression, and the ability to relapse after cancer therapies.^87^ Inflammatory cells and cytokines demonstrably support CSCs. In the liver, the predetermining factors that contribute to the disruption of the liver microenvironment generate the niche (or niches) suitable for CSCs survival and expansion. In support of this, transplantation of hepatic progenitor cells gave rise to cancer only when they were introduced into chronically injured livers.^58^ The IL-6/STAT3 pathway is an important pathway promoting the CSC expansion. Cells with progenitor cell feature quiescently resided in dysplastic lesions for a long time and did not undergo malignant transform until they acquire autocrine IL-6 signaling.^58^ IL-6 treatment induced expansion of the CD133-positive cancer progenitor population in vitro.^77^ A recent report also demonstrated that TAMs enhanced human HCC CSC phenotype by IL-6 (from TAM) /STAT3 (in cancer cells) paracrine signaling pathway.^114^ IL-6/STAT3 signaling also contributed to hepatic progenitor cell expansion in HBx-transgenic mice.^115^ Additionally, IL-6, TNFα, or TGFβ1-treated HepaRG-tdHep cells showed retrodifferentiate to the hepatic progenitor cell-like cells.^116^ Consistent with it, our lab also found TGFβ gave rise to TICs and promote HCC development.^117^ Another report from our lab found a long noncoding RNA lnc-DILC mediated the crosstalk between TNF-α/NF-κB signaling and autocrine IL-6/STAT3 cascade and thus connected hepatic inflammation with HCC CSC expansion.^118^

In addition to IL-6, a recent report demonstrated that lymphotoxin β (LTβ) is an important cytokine for the HCC progenitor cells (HPC) appearance and maturation in the ectopic lymphoid structures (ELSs), and is associated with the egression of hepatic progenitor cell out of the ELSs and tumor formation.^102^ Another important microenvironmental signaling pathway associated with CSCs survival and expansion within HCC during liver injury is the TWEAK ((tumor necrosis factor (TNF)­like weak inducer of apoptosis)–FN14 (also known as TNFRSF12A)) pathway.^119^ Infiltrating macrophages could be identified as an important source of TWEAK.^120^ Furthermore, the cell fate decision of stem or progenitor cells of the liver was shown to be dependent on the type of liver injury (biliary cells or hepatocytes damage) as well as on the interaction between stem or progenitor cells with activated myofibroblasts or macrophages within the liver.^121^

### Activating invasion and metastasis

Around 90% of cancer mortality is caused by tumor metastasis. The metastatic process consists of a series of events: cell detachment from the primary tumor mass, migration into and transport along the bloodstream, and finally tumor cell arrest and proliferation within the distant tissue.^122^ Numerous evidences show that inflammatory cells and mediators they produced substantially facilitate tumor cell invasion, extravasation, and metastatic outgrowth,^123^ but the underlying mechanisms are far from elusive. Cancer cells with metastatic potential usually undergo the epithelial–mesenchymal transition (EMT), wherein they depolarize, lose cell–cell contacts, and acquire mesenchymal cell characteristics such as enhanced motility, invasion, and resistance to apoptotic stimuli.^124^ TGFβ, though inhibiting tumor growth at the early stages, is a strong inducer of EMT.^125^ TGFβ1 promotes EMT through downregulation of E-cadherin, a major component of the epithelial adherent junctions, and upregulation of the E-cadherin repressor *SNAIL* and PDGF intracellular signaling.^126^ A recent report showed that lncRNA-ATB mediates the role of TGFβ in inducing HCC EMT and promoting metastasis.^127^ Other cytokines such as TNFα,^128^ IL-6^114^, IL-8^86^, CCL22,^129^ HMGB1^34^ also promote HCC cell invasiveness by enhancing EMT and/or upregulating the production and activity of MMPs.^34^ MMP-9-expressing macrophages were involved in matrix remodeling and degradation at the invasive front of murine HCC.^130^ Thanks to their proteolytic activity, MMP-9, and MMP-2 promote ECM-stored growth factors mobilization, including VEGF, thus favoring angiogenesis in HCC.^91,131^ Following EMT, the rest steps of metastasis such as intravasation, survival in the circulation, extravasation and successful colony also require the inflammatory machine.^122,132^ However, as most of these data were from tumors of other organs, whether HCC cells also deploy these mechanisms needs to be elucidated. To do so, a more HCC-specific metastasis model is needed to mimic the metastasis process in vivo.

### Inducing genome instability

Increased genome instability accompanied by genomic mutagenesis has been demonstrated to be a crucial driving force of liver cancer.^133^ Inflammation can increase the rates of DNA damage while compromise DNA repair mechanisms that are required to maintain genomic integrity, leading to increased genomic instability and promoting the initiation of neoplastic transformation. In an inflammation-associated HCC model, the Mdr2 knockout mice, chronic inflammation was associated with DNA double-string break, DNA damage response and genome instasbility.^134^ The mechanisms by which inflammatory mediators induce somatic mutation are divers, which may include direct or indirect function, through a cell-intrinsic or a cell-extrinsic manner. First, ROS and NOS (released by tissue neutrophils and macrophages, or produced intracellularly in premalignant cells induced by inflammatory cytokines) cause DNA strand breaks, single base mutations or more complex DNA damage, thus break the genes that control cell growth or differentiation. They can also cause post-translational modification of proteins that control cell cycle or survival.^19,135^ Furthermore, intrahepatic chronic hypoxia, which augments ROS production, may occur during the inflammatory and fibrotic processes that characterize several CLDs.^136^ Hypoxic cells may have decreased DNA repair and increased chromosomal instability.^137^ Secondly, several cytokines, including TNFα, IL-1β, and TGFβ, have been shown to induce ectopic expression of activation-induced cytidine deaminase (AID), a member of the DNA and RNA cytosine deaminase family, which introduces mutations in crucial cancer-associated genes such as *TP53* and *MYC* and thereby promotes hepatocarcinogenesis.^138^ Thirdly, inflammatory cytokines can inhibit the DNA repair.^139^ Last but not the least, inflammatory cytokines such as IL-6 may promote the cells harboring DNA damage division, thus lead to the increased mutagenesis and genome instability, as shown in a recent study using the Mdr2-deficient mice.^140^ Collectively, chronic inflammation cause deregulated DNA damage response and induce genomic instability, which leads to loss of tumor suppressors and/or oncogene activation, thereby set the first stage of hepatocarcinogenesis while inflammatory cytokines promote mutated cell expansion. In addition, DNA damage response can amplify inflammatory responses, thus provide a vicious cycle that promotes the genomic instability.^68^

## Conclusion

In the past few decades, accumulating data in HCC study have made the epidemiology and etiology of HCC well established. However, its molecular pathogenesis is still poorly understood. As a consequence, mechanism-based effective therapies for HCC are few. HCC occurs predominantly in patients with CLDs, which appears as hepatitis, fibrosis, and cirrhosis,^141^ suggesting HCC is a prototype of inflammation-associated cancer. While a controlled inflammation may be beneficial to the host, increasing data showed that deregulated inflammation is a recognized hallmark of cancer that substantially contributes to the development and progression of malignancies.^14^ Nonresolving inflammation in the liver can be initiated and propagated by DAMPs released by damaged hepatocytes or the products of the gut microbe. The intrinsic pathway of nonresolving inflammation is orchestrated by genetic events in the tumor cells without initiation by prior inflammation. The inflammation has been shown to contribute to several hallmarks of HCC, such as promoting proliferative and survival signaling, inducing angiogenesis, evading immune surveillance, supporting CSCs, inducing genome instability, and activating invasion and metastasis. Thus, targeting inflammation—such as targeting the gut-live axis thus dampening immune cell activation; targeting the inflammatory cells, e.g., TAM, monocyte, CD4^+^ T cells, CD8^+^ T cells, DCs; targeting the key inflammatory pathways—is believed to be an attractive therapeutic strategy in the management of HCC. Indeed, the use of nonsteroidal anti-inflammatory drugs decrease the incidence and/or recurrence of HCC.^16–18^ The combination therapy of zoledronic acid and sorafenib to treat advanced HCC is being evaluated in phase II studies (NCT01259193). Much therapeutics has been designed to block cytokine receptors and downstream signaling pathways such as receptor kinases and STAT3 to inhibit inflammation-driven protumoral signal. Sorafenib is one of such inhibitors and has been approved by FDA for treatment of advanced HCC.^8,9^ More inhibitors are in different stages of clinical trials.^89,142,143^ Supplementary Table 1 summarized the current completed randomized clinical trials in HCC from the website ClinicalTrials.gov. Moreover, as HCC is an immunogenic liver lesion that expresses shared tumor antigens (tumor-associated antigens) and private neo-antigens arising from specific gene mutations, immunotherapy for HCC is an appealing option to reduce the risk of relapse by eliminating micrometastatic residual disease after surgical or percutaneous ablation.^144^ However, there are some challenges for translating basic research into clinical therapeutics. First, it is quite challenging to accurately and precisely duplicate human HCC development in relevant animal models. For example, follow-up in human with long-term inflamed liver conditions has revealed the sequential change of the liver fibrosis, cirrhosis, preneoplastic niches, HCC initiation, and eventually progresses and metastasizes. This process usually takes a long time period. By contrast, in most mouse models, progression from inflammation to cirrhosis does not occur-dysplastic change usually appears subsequent to inflammation, and finally leads to HCC development. A different conclusion may be made for one issue based on the different mouse model. Second, the tumor-host immune response is so complicated that the key mechanisms underlying this response remain far from fully understood. Cancer-related inflammation is not only a local immune response, but also a systemic immune response. Opposing effects of local and systemic inflammation create the biggest challenge to HCC treatment.^145^ Last but not least, some potential therapeutics may have some unexpected side effects. For example, deregulation of the PI3K/Akt/mTOR pathway was increasingly implicated in HCC carcinogenesis.^146^ However, a recent report showed that combined deletion or pharmacological inhibition of AKT1 and AKT2 isoforms led to liver injury, inflammation, and hepatocarcinogenesis.^147^

Collectively, inflammation plays crucial roles in hepatocarcinogenesis. Targeting inflammation already present a logical avenue for HCC treatment. There are also a lot of challenges for the successful translation from bench to bedside.

## Supplementary information

Supplementary Table 1
