# Supplementary material for: Role of nonresolving inflammation in hepatocellular carcinoma development and progression
Source: NPJ Precis Oncol. 2018 Feb 23;2:6. doi: 10.1038/s41698-018-0048-z (PMC5871907; doi:10.1038/s41698-018-0048-z)
Supplement: Supplementary file 1 — Supplementary Table 1 [file 41698_2018_48_MOESM1_ESM.pdf]

NCT NO	Drug	Other treatment	Trial Design	Trial Phase	Start	End	Last Update posted	No of Enrolled Patients	Incidence of Recurrence and Metastasis After Hepatectomy	OS(nonths) Overall Survival	RFS(month) Recurrence Free Survival	PFS (months) Progression-Free Survival	ORR(%) Objective Response Rate	TTR(month) Time to Recurrence	TTSP(month) Time to Systemic Progression	TTP (month) Time to Tumor Progression	DR (month) duration of response	DCR(%)	CBR Percentage of Participants With Overall Clinical Benefit Response	Reference (PMID)	Target Pathways
NCT01210495	Axitinib (AG-013736)<br>VS. Placebo	Best Supportive care (BSC)	Randomized<br>Parallel assignment<br>Double Blind	2	Dec 2010	Dec 2016	03-28-2017	224		12.7(10.2-14.9)<br>9.7(5.9-11.8)<br>p=0.2872		3.6(2.3-4.6)<br>1.9(1.9-3.5)<br>p=0.0039	9.7(5.3-16)<br>2.9(0.4-10.2)<br>p=0.0914			3.7(2.8-5.6)<br>1.9(1.9-3.6)<br>p=0.006	6.4(3.7-9.3)<br>N/A		31.3(23.6-39.9)<br>11.8(5.2-21.9)<br>p=0.0025	26386123	VEGFR, PDGFR
NCT01140347	Ramucirumab DP (IMC-1121B)<br>VS. Placebo	BSC	Randomized<br>Parallel assignment<br>Double Blind	3	October 2010	March 2015	28-Dec-15	565		9.17 (8.05 to 10.64)<br>7.62(6.01-9.33)<br>p=0.1391		2.79 (2.69 to 3.94)<br>2.10 (1.58 to 2.69)<br>p<0.0001	7.1 (4.6 to 10.7)<br>0.7 (0.2 to 2.5)<br>p<0.0001			3.48 (2.76 to 4.47)<br>2.63 (1.58 to 2.76)<br>p<0.0001				27657674<br>26095784	VEGFR2
NCT01232296	Dovitinib<br>VS. Sorafenib		Randomized<br>Parallel assignment<br>Open Label	2	July 2011	April 2014	04-Dec-15	165		33 (25.7 to 39)<br>39.4(22.6 to 56.1)						17.6 (12.3 to 18.4)<br>17.9 (12.3 to 18.9)					FGFR3, VEGFR, PDGFR3, Flt3,<br>c-Kit (Dovitinib)
NCT01203787	Sorafenib Standard Dosing Regimen<br>VS. Sorafenib Ramp-Up Regimen		Randomized<br>Parallel assignment<br>Open Label	4	Dec 2010	March 2014	05-Mar-15	120													Raf, VEGFR2/PDGFRβ
NCT00699816	Immuncell-LC<br>VS. Control		Randomized<br>Parallel assignment<br>Open Label	3	Jul 2008	Nov 2012	9-24-2015	230		N/A<br>N/A<br>p=0.008	44(42.46-44.54)<br>30(28.25-31.75)<br>p=0.01									25747273	
NCT01379521	Everolimus<br>VS. Placebo	TACE	Randomized<br>Parallel assignment<br>Quadruple blind	2	Jun 2011	June 2015	5-3-2017	65		29.9 (12.0 to N/A)<br>21.7 (19.4 to 27.9)						6.3(4.3-6.6)<br>6.4(4-8.9)					mTOR
NCT00699374	Sunitinib Malate<br>VS. Sorafenib		Randomized<br>Parallel assignment<br>Open Label	3	Jul 2008	Dec 2011	1-14-2013	1075		34.3 (31.9 to 39.9)<br>43.9 (38.1 to 48.7)<br>p=0.9993		15.3 (12.1 to 17.7)<br>12.6 (12.1 to 17.3)<br>p=0.8857				17.7 (13.6 to 18.0)<br>15.4 (12.3 to 18.1)<br>p=0.8459				24081937<br>20203173	VEGFR2, PDGFRβ,<br>c-Kit, Flt3
NCT00881751	Bevacizumab + Erlotinib<br>VS. Sorafenib		Randomized<br>Parallel assignment<br>Open Label	2	Mar 2009	Feb 2017	11-Sep-17	95		8.55 (7.00 to 13.9)<br>8.55 (5.69 to 12.2)		4.37 (2.99 to 7.36)<br>2.76 (1.84 to 4.80)									VEGF(Bevacuzumab)<br>EGFR(Erlotinib)
NCT00049322	Bevacizumab<br>VS. Control	TACE	Randomized<br>Crossover assignment<br>Open Label	2	June 2003	Feb 2012	15-Mar-16	30													VEGF
NCT01770431	Huaier Granule<br>VS. Blank Control		Randomized<br>Parallel assignment<br>Double Blind	4	Aug 2011	Aug 2016	Oct-2-2017	1044	37.6%<br>50.9%<br>p=0.0001	75.5±1.15 (SE)<br>68.5±1.89 (SE)<br>p<0.0001											modulation of various pathways in carcinogensis<br>and angiogenesis
NCT00108953	Sorafenib + Doxorubicin<br>VS.<br>Placebo+Doxorubicin		Randomized<br>Parallel assignment<br>Double Blind		Apr 2005	Apr 2008	31-Oct-14	96		13.93(10.57-N/A)<br>6.63(4.93-10.06)<br>p=0.007		8.07(4.67-10.4)<br>2.83(2.37-5.73)<br>p=0.018				8.77(4.87-12.8)<br>4.9(2.2-8.13)<br>p=0.016					Raf, VEGFR2/PDGFRβ
NCT00692770	Sorafenib<br>VS. placebo		Randomized<br>Parallel assignment<br>Quadruple Blind	3	Aug 15, 2008	Nov 28, 201[illegible]	16-Oct-17	1114		NA<br>NA<br>p=0.484742	33.8(27.97-44.63)<br>34.2(28.03-39.5)<br>p=0.258329			18.53(20.8-NA)<br>36.3(30.77-42)<br>p=0.121383						26361969	Raf, VEGFR2/PDGFRβ
NCT01035229	Everolimus<br>VS. Placebo	BSC	Randomized<br>Parallel assignment<br>Double Blind	3	April 2010	Oct 2013	22-Sep-16	546		7.56 (6.70 to 8.74)<br>7.33 (6.28 to 8.74)						2.96 (2.79 to 4.01)<br>2.60 (1.48 to 2.83)		56.1<br>45.1		25058218	mTOR
NCT00105443	Sorafenib<br>VS.Placebo		Randomized<br>Parallel assignment<br>Double Blind	3	March 2005	Nov 2008	31-Oct-14	602		10.8(9.53 to 13.5)<br>8.03 (6.87 to 9.2)<br>p=0.00583					4.2(3.5-4.2)<br>4.93(4.3-6.4)<br>p=0.7676	5.6(4.2-7)<br>2.87(2.73-4)<br>p=0.000007				18650514	Raf, VEGFR2/PDGFRβ
NCT00492752	Sorafenib<br>VS.Placebo		Randomized<br>Parallel assignment<br>Triple Blind	3	Oct 2005	Jul 2009	16-Apr-14	226		6.6 (5.63 to 7.67)<br>4.23 (3.8 to 5.53)<br>p=0.14144					3.5 (2.83 to 4.3)<br>3.43 (2.43 to 4.13)<br>p=0.497537	2.8 (2.67 to 3.63)<br>1.38 (1.37 to 1.57)<br>p=0.000537				19095497<br>22240282	Raf, VEGFR2/PDGFRβ
NCT00987935	BIBF 1120<br>VS. Sorafenib		Randomized<br>Parallel assignment<br>Open Label	2	Oct 2009	Jan 2016	10-Mar-16	134		10.18(5.19 to 16.85)<br>10.71 (7.29 to 16.66)		2.66 (0.99 to 5.55)<br>3.71 (1.77 to 7.36)				2.76 (0.99 to 5.55)<br>3.71(1.77 to 7.36)					
NCT00855218	Sorafenib<br>VS. Placebo	TACE	Randomized<br>Parallel assignment<br>Quadruple Blind	2	March 2009	Feb 2013	18-Aug-17	307		NA(18.47-N/A)<br>NA(18.73-N/A)<br>p=0.295						5.63(5.53-7.3)<br>5.53(3.77-5.6)<br>p=0.072				26809111	Raf, VEGFR2/PDGFRβ

Table 1. Complete randomized clinical trials targeting inflammation in HCC

Interventional clinical trials in HCC that match the following criteria in ClinicalTrilas.gov were selected: (1) terminated or completed studies, (2) with results, (3) phase 2 or 3 or 4, (4) drugs targeting inflammation-associated pathways, (5) allocation: randomized, and (6) intervention model: parallel or crossover design.
